# *Microbacterium* Cluster EA Bacteriophages: Phylogenomic Relationships and Host Range Predictions

**DOI:** 10.3390/microorganisms11010170

**Published:** 2023-01-10

**Authors:** Mark Milhaven, Cyril J. Versoza, Aman Garg, Lindsey Cai, Sanjana Cherian, Kamalei Johnson, Kevin Salas Perez, Madison Blanco, Jackelyn Lobatos, Corinne Mitra, Maria Strasser, Susanne P. Pfeifer

**Affiliations:** 1 Center for Evolution and Medicine, School of Life Sciences, Arizona State University, Tempe, AZ 85281, USA; 2 School of Life Sciences, Arizona State University, Tempe, AZ 85281, USA

**Keywords:** bacteriophage, cluster EA, comparative genomics, host range

## Abstract

Bacteriophages are being widely harnessed as an alternative to antibiotics due to the global emergence of drug-resistant pathogens. To guide the usage of these bactericidal agents, characterization of their host specificity is vital—however, host range information remains limited for many bacteriophages. This is particularly the case for bacteriophages infecting the *Microbacterium* genus, despite their importance in agriculture, biomedicine, and biotechnology. Here, we elucidate the phylogenomic relationships between 125 *Microbacterium* cluster EA bacteriophages—including members from 11 sub-clusters (EA1 to EA11)—and infer their putative host ranges using insights from codon usage bias patterns as well as predictions from both exploratory and confirmatory computational methods. Our computational analyses suggest that cluster EA bacteriophages have a shared infection history across the *Microbacterium* clade. Interestingly, bacteriophages of all sub-clusters exhibit codon usage preference patterns that resemble those of bacterial strains different from ones used for isolation, suggesting that they might be able to infect additional hosts. Furthermore, host range predictions indicate that certain sub-clusters may be better suited in prospective biotechnological and medical applications such as phage therapy.

## 1. Introduction

First discovered in the early 1900s, bacteriophages (i.e., viruses that infect, and ultimately kill, bacteria) are the most abundant biological entities on earth, with an estimated 10^31^ viral particles [1]. Yet, despite their abundance across environmental systems and their important impact on the evolution and community dynamics of the bacterial biosphere, much bacteriophage diversity remains uncharacterized to date.

Due to their host specificity and bactericidal nature, lytic bacteriophages are promising agents in many applications ranging from agriculture (e.g., to treat crops infected with pathogenic bacteria [2]) to biomedicine (e.g., to develop therapies for patients infected with multi-drug resistant *Microbacterium* strains [3]) to food safety (e.g., to prevent zoonotic pathogens in poultry, pork, beef, and fish [4]) and to wastewater treatment (e.g., to prevent sludge foaming [5]). Hence, gaining a better understanding of the genomic diversity of bacteriophages and the bacterial strains that they are able to infect is an important direction of current scientific research.

To aid in this endeavor, undergraduate researchers of the Science Education Alliance—Phage Hunters Advancing Genomics and Evolutionary Science (SEA-PHAGES) project have isolated, sequenced, and genomically characterized nearly 4000 bacteriophages over the last decade [6]. Of these, 514 bacteriophages infect Gram-positive, rod-shaped aerobic *Microbacterium* hosts that have been isolated from a variety of sources, including humans, where they can cause opportunistic infections in immuno-compromised individuals ([7] and references therein), as well as food [8], soil [9], and plants [10,11] (for additional details, see the Actinobacteriophage Database: https://www.phagesdb.org (accessed on 29 November 2022) [12]).

Based on their nucleotide similarity, bacteriophages infecting microbacterial hosts can be classified into 15 clusters (clusters EA–EM and GA–GF), many of which are further separated into sub-clusters whose members share common genomic characteristics [13]. Among these 15 clusters, the cluster with the largest number of members, cluster EA (148 members), consists almost exclusively (~96%) of obligatory lytic bacteriophages isolated from *M. foliorum* NRRL B-24224, with the exception of bacteriophages Ixel, Leafus, Mercedes, and Nebulous isolated from *M. liquefaciens* LMG 16120 [14], Theresita isolated from *M. natoriense* ATCC BAA-1032 [13], and WilliamStrong isolated from *M. paraoxydans* NRRL B-14843. Consequently, the complete host range of bacteriophages from this representative cluster remains relatively poorly understood.

Bacteriophage isolation and cultivation remains the gold standard for characterizing bacteriophage-host interactions. At the “School of Life Sciences”; same time, experimental approaches are cost-, labor-, and time-intensive, thus limiting the number of studies that can feasibly be performed in many laboratories, especially as part of course-based undergraduate research programs with often limited budgets. However, recent advances in high-throughput sequencing technologies as well as associated bioinformatic methods have provided an alternative means to computationally predict bacteriophage–host interactions based on genomic features shared between bacteriophages and their hosts due to their co-evolution (see review of [15]). For example, as obligate parasites [16], bacteriophages require the bacterial host machinery to synthesize proteins—a strategy that is generally most efficient when the codon usage patterns of the bacteriophage closely match those of its host [17]. Consequently, patterns of codon usage bias (i.e., the preferential usage of synonymous codons) can provide important insights into the evolutionary relationships between bacteriophages and their hosts as well as regions of horizontal gene transfer (see review of [18]). To obtain a more complete picture, this information can then be combined with genome-wide levels of virus–host similarity, for example, based on the frequencies of different oligonucleotides in the virus and host genomes [19].

As part of a course-based undergraduate research experience at Arizona State University, we here characterize the phylogenomic relationships and computationally infer the putative host ranges of all cluster EA bacteriophages known to date. These novel insights will aid the future design of experimental assays to investigate bacteriophage–host relationships and may elucidate potential applications of cluster EA bacteriophages.

## 2. Materials and Methods

Publicly available whole-genome sequence data for 125 cluster EA bacteriophages were downloaded from NCBI GenBank (for accession numbers, see Supplementary Table S1) to characterize their phylogenomic relationships. In brief, a whole-genome multiple-sequence alignment (MSA) was generated between the bacteriophage genomes using the fast Fourier transform (FFT-NS-2) in MAFFT v.7.402 [20], pairwise average nucleotide identities (ANIs) calculated using the genome comparison tool in DNA Master v.5.23.6 and plotted in R v.4.0.2 [21] using the ggplot2 package [22]. In addition, a neighbor-joining tree was built from the MSA in MEGA v.11 [23] using a phylogeny test with 10,000 bootstrap replicates and subsequently visualized using FigTree v.1.43 (http://tree.bio.ed.ac.uk/software/figtree/ (accessed on 29 November 2022)). For each group in the neighbor-joining tree, nucleotide sequence relatedness was analyzed using dot plots from Gepard v.2.1.0 [24].

To obtain insights into which *Microbacterium* species each bacteriophage might be able to infect, COUSIN v.0.4 [25] was used to determine the COdon Usage Similarity INdex (COUSIN_59_) for each of the 125 cluster EA bacteriophages (Supplementary Table S1) across 14 putative microbacterial host species (Supplementary Table S2), including *M. foliorum* B-24224 (i.e., the experimentally validated host for the majority of the isolated cluster EA bacteriophages). Following Howell, Versoza et al. [26], host ranges were predicted using both exploratory and confirmatory methods—PHERI v.0.2 [27] and WIsH v.1.1 [19], respectively. PHERI is a machine-learning-based tool that capitalizes on protein sequence similarity, while WIsH utilizes the oligonucleotide frequency profiles of bacteriophages to predict prospective hosts. Thereby, likelihoods are calculated under trained Markov models, each corresponding to a potential host genome, and the model that produces the highest likelihood is determined to be the likely host. All software was executed using default settings.

## 3. Results

**Figure 1:**
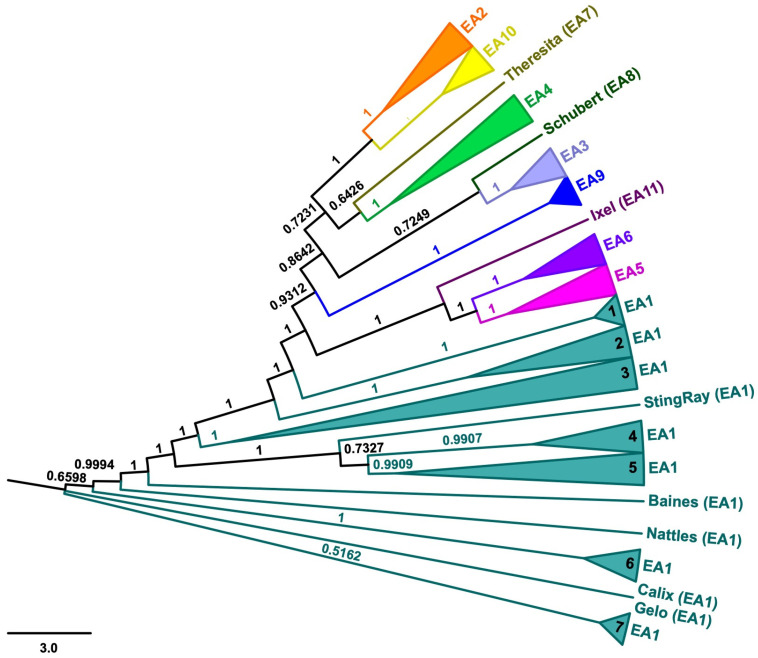
Neighbor-joining tree. Neighbor-joining tree generated using a multiple-sequence alignment of 125 cluster EA bacteriophage genomes (Supplementary Table S1) with 10,000 bootstrap replicates. Colors highlight bacteriophage membership in sub-clusters EA1–EA11. Numbers on the branches indicate bootstrap values.

Comparative genomic analyses on 125 cluster EA bacteriophages—including 88 members of sub-cluster EA1, 7 of sub-cluster EA2, 3 of sub-cluster EA3, 8 of sub-cluster EA4, 7 of sub-cluster EA5, 4 of sub-cluster EA6, 1 of sub-cluster EA7, 1 of sub-cluster EA8, 2 of sub-cluster EA9, 3 of sub-cluster EA10, and 1 of sub-cluster EA11 (Supplementary Table S1)—demonstrated high levels of sequence relatedness in both the dot plot analysis (Supplementary Figure S1 and Supplementary Table S3) and the pairwise ANIs calculated between the cluster EA bacteriophages (Supplementary Figure S2), with groupings in the neighbor-joining tree (Figure 1) in agreement with previous cluster assignments [13]. Interestingly, however, sub-cluster EA1 appears further sub-divided into seven distinct groups as well as five singletons (Baines, Calix, Gelo, Nattles, and StingRay).

**Figure 2:**
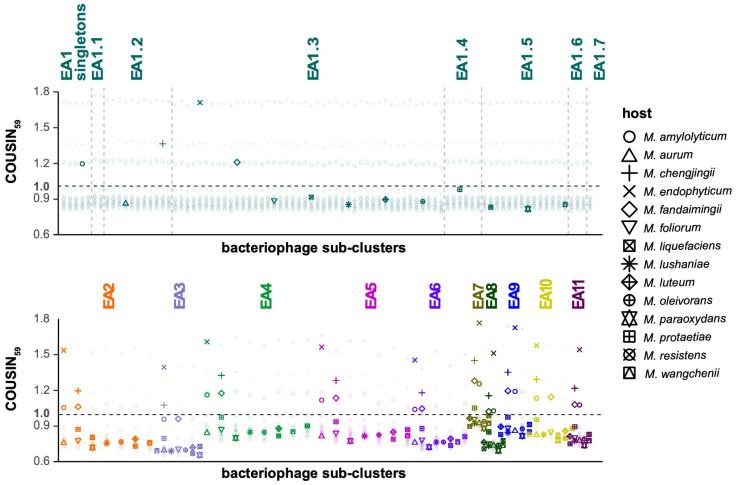
Codon usage bias. COdon Usage Similarity INdex (COUSIN_59_) of 125 cluster EA bacteriophages (Supplementary Table S1) across 14 potential *Microbacterium* host species (Supplementary Table S2), ordered by sub-cluster assignment of the bacteriophages. A COUSIN_59_ score of 1 (horizontal black dashed line) indicates that a bacteriophage–host pair exhibits similar codon usage preferences. Colors highlight bacteriophage membership in sub-clusters EA1–EA11; vertical gray dashed lines indicate sub-cluster EA1 groupings in the neighbor-joining tree (Figure 1); shapes highlight *Microbacterium* species; individual bacteriophage–host COUSIN_59_ scores are shown as transparent symbols and mean COUSIN_59_ scores for each potential host in a sub-cluster are shown as non-transparent symbols.

**Figure 3:**
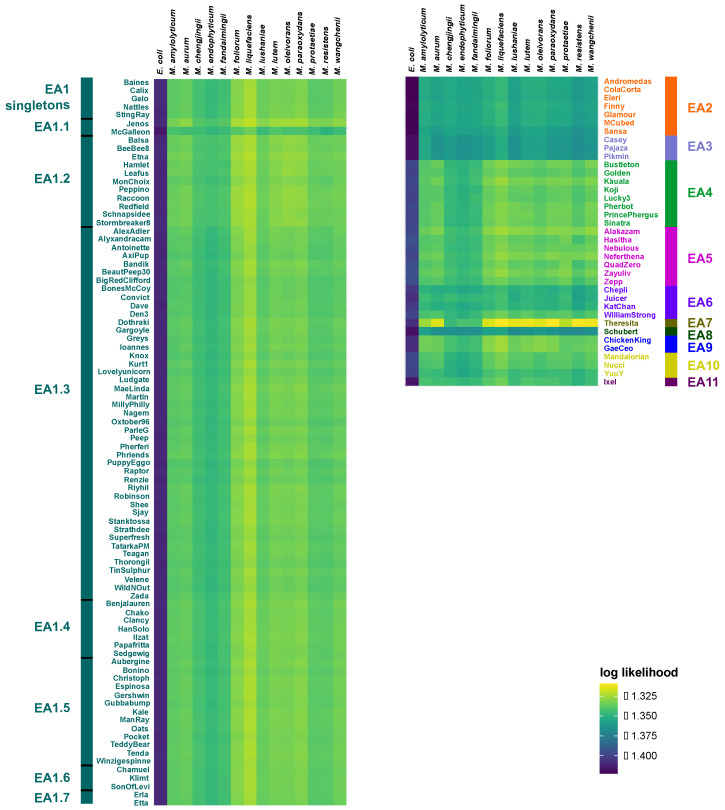
Predicted host ranges. Heatmap of log likelihoods of bacteriophage–host pairs, including 125 cluster EA bacteriophages (Supplementary Table S1), 14 potential *Microbacterium* host species (Supplementary Table S2), and *Escherichia coli* as a negative control as predicted by WIsH. Colored names and bars highlight bacteriophage membership in sub-cluster EA1–EA11 groupings in the neighbor-joining tree (Figure 1); higher values in the heatmap correspond to more likely bacteriophage-host interactions.

Patterns of codon usage bias among the 125 bacteriophages show a strong agreement in codon usage preferences (CUPrefs) across the 14 potential *Microbacterium* hosts (Figure 2). Of particular note, bacteriophages of all sub-clusters more closely resemble CUPrefs of bacterial strains different from those used for isolation, suggesting that they might be able to infect additional hosts. Specifically, bacteriophages from sub-clusters EA1, EA4–5, and EA8–10 exhibit CUPrefs that are most similar to *M. protaetiae*, EA2, EA6, and EA11 to *M. amylolyticum*, EA3 to *M. fandaimingii*, and EA7 to *M. liquefaciens*. Despite this, *M. foliorum* (the host used for isolation for the majority of the cluster EA bacteriophages) and *M. liquefaciens* (used to isolate Ixel, Leafus, Mercedes, and Nebulous) are consistently predicted as the most likely bacterial hosts across the cluster EA bacteriophages based on their similarity in oligonucleotide frequency profiles (Figure 3). Notably, members of the EA2, EA3, and EA8 (Schubert) sub-clusters have relatively low log likelihoods compared to the remainder of the sub-clusters, suggesting that more suitable hosts might exist outside of the 14 strains tested. This observation is further supported by the results of the exploratory host prediction analysis which highlighted that the members of these sub-clusters are also likely to infect hosts of the *Burkholderia*, *Mycolicibacterium*, and *Rhizobium* genera (Supplementary Table S4). In addition, these analyses suggest that Theresita—a sub-cluster EA7 singleton—likely exhibits a much broader host range across *Microbacterium* species.

## 4. Discussion

Bacteriophages are utilized for a variety of applications in agriculture, biomedicine, biotechnology, and diagnostics [28]. To effectively implement these bactericidal agents, it is crucial to understand phylogenomic relationships between bacteriophages and to ascertain their host ranges. Comparative genomic analyses confirmed previously established cluster memberships of the analyzed bacteriophages, yet the additional groupings of cluster EA1 bacteriophages in the neighbor-joining tree (Figure 1) suggest that relationships cannot be fully resolved at the sub-cluster level. With regards to host specificity, the reported codon usage bias (indicated by the COUSIN_59_ index; Figure 2) demonstrates a shared pattern across bacteriophages, with clustering of CUPrefs scores in accordance with sub-cluster assignment. Strikingly, cluster EA bacteriophages harboring a median GC content of 63.4% (Supplementary Table S1) exhibit codon usage preferences that are similar to those found in bacterial strains with GC contents ranging from 63.3% (*M. fandaimingii*) to 68.3% (*M. liquefaciens*) (Supplementary Table S2). In contrast, patterns of codon usage bias across bacteriophages are distinctly different from the microbial strains with both the lowest and highest GC content—*M. chengjingii* (61.8%) and *M. endophyticum* (61.9%) as well as *M. wangchenii* (70.6%), *M. lushaniae* (70.7%), and *M. resistens* (71.3%)—suggesting that cluster EA bacteriophages are more likely to have shared an evolutionary infection history with the remainder of the host species. Consistent with the patterns of codon usage preference, and based on the oligonucleotide frequency similarity between bacteriophage and host genomes, the confirmatory tool WIsH predicted host species with low and high GC content to be less likely hosts of cluster EA bacteriophages. At the same time, both confirmatory (WIsH) and exploratory (PHERI) analyses predicted that several additional members of the *Microbacterium* genera might be potential hosts for cluster EA bacteriophages. These host range predictions are in agreement with experimentally validated results of successful *M. foliorum*, *M. liquefaciens*, and *M. paraoxydans* infections (Supplementary Table S1). While the majority of bacteriophages exhibit similar likelihood estimates across bacterial hosts, the observation of elevated log likelihood values suggests that certain bacteriophages should be favored in antimicrobial strategies. For example, a phage cocktail comprised of a combination of sub-cluster EA1 and EA7 members would allow for a broadening of host range when dealing with different *Microbacterium* species and strains. As such, bacteriophages of these sub-clusters will be important candidates for follow-up experimental validations of host specificity to aid future applications using bacteriophages of cluster EA as bactericidal agents.

## Data Availability

Genomic data for cluster EA bacteriophages and putative bacterial host species can be downloaded from NCBI GenBank using the accession numbers provided in Supplementary Table S1 and Supplementary Table S2, respectively.

## Supplementary Materials

The following supporting information can be downloaded at: https://www.mdpi.com/article/10.3390/microorganisms11010170/s1, Figure S1: Dot plots; Figure S2: Average nucleotide identities; Table S1: Cluster EA bacteriophages included in the comparative analyses [10,13,14,29,30,31,32,33,34,35,36]; Table S2: Host bacteria included in the comparative analyses [10,37,38,39,40,41,42,43,44,45,46,47,48]; Table S3: Average nucleotide identities of representative cluster EA bacteriophages used in the dot plot analysis; Table S4: Exploratory host range prediction [27].
