# Supplementary material for: Microbacterium Cluster EA Bacteriophages: Phylogenomic Relationships and Host Range Predictions
Source: Microorganisms. 2023 Jan 10;11(1):170. doi: 10.3390/microorganisms11010170 (PMC9863963; doi:10.3390/microorganisms11010170)
Supplement: Supplementary file 1 [file microorganisms-11-00170-s001.zip › microorganisms-2098370-SuppFigures.pdf]

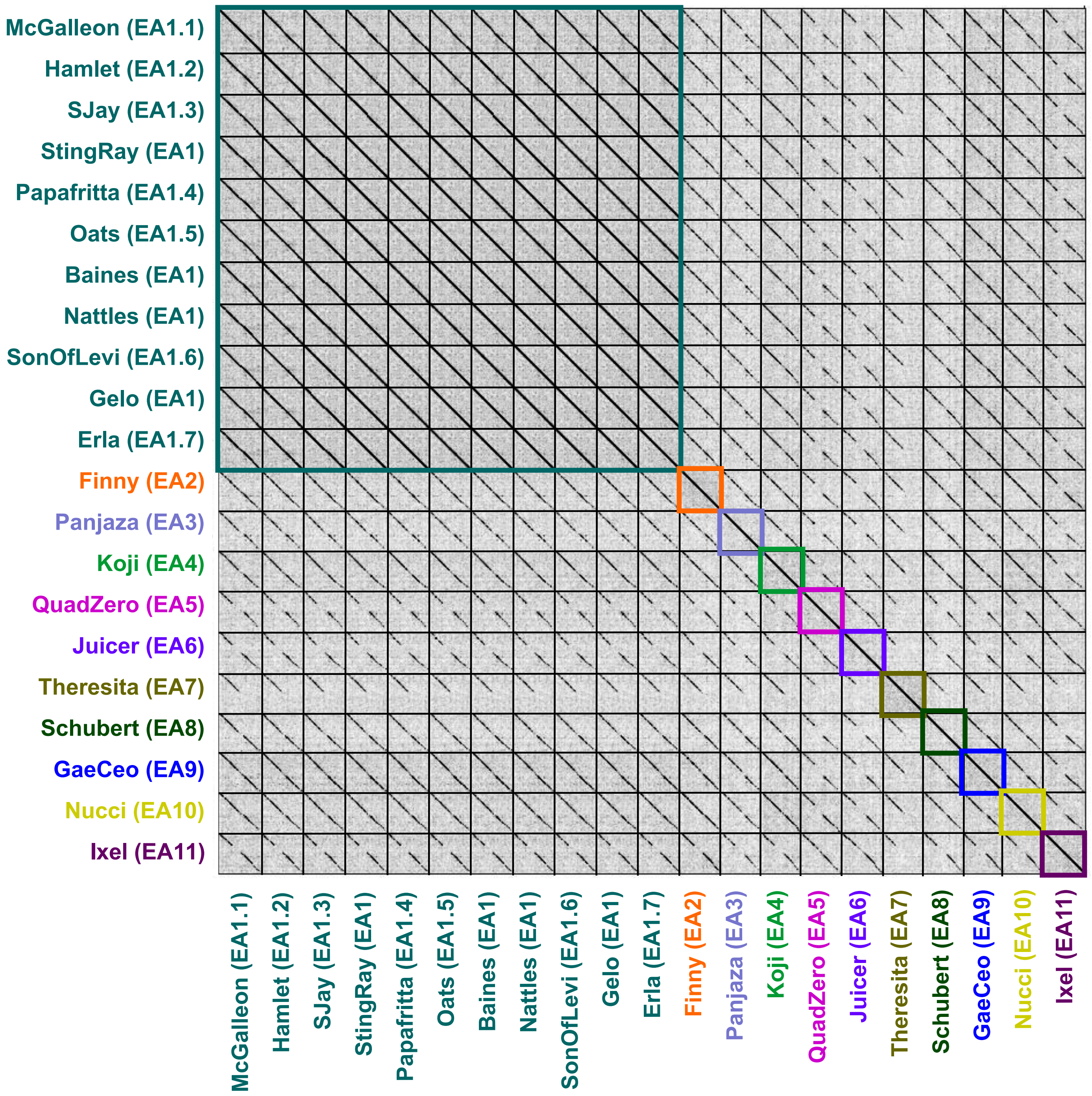

Supplementary Figure S1. Dot plots of representative cluster EA bacteriophages from each of the groupings shown in the neighbor-joining tree (Figure 1). Sub-clusters (EA1 to EA11) are indicated by colors. Additional details on these bacteriophage genomes can be found in Supplementary Table S1.

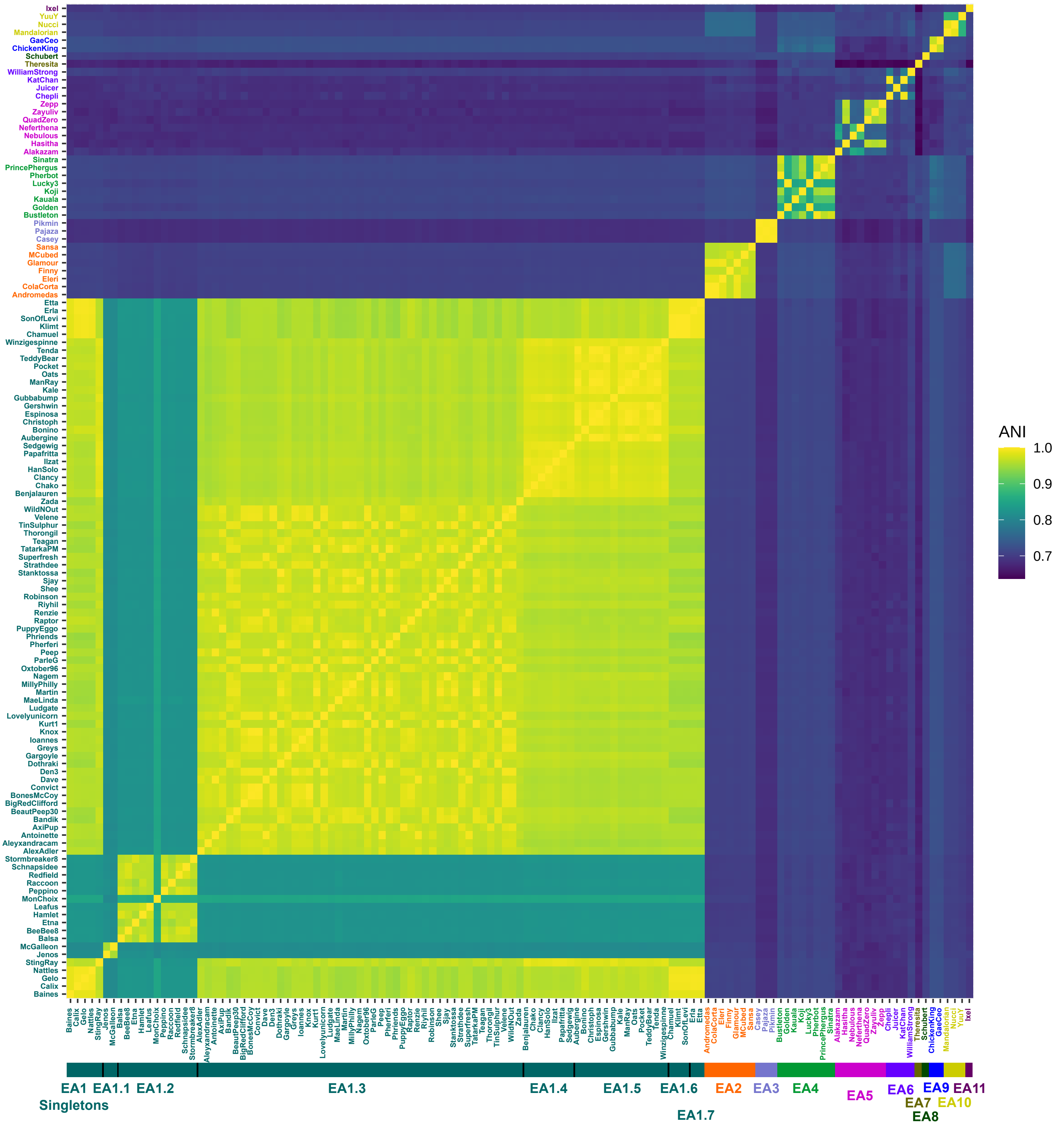

Supplementary Figure S2. Average nucleotide identities (ANIs) of closely-related cluster EA bacteriophages from each of the groupings shown in the neighbor-joining tree (Figure 1), with sub-clusters (EA1 to EA11) indicated by colors. Additional details on these bacteriophage genomes can be found in Supplementary Table S1.
