# Supplementary material for: Microbacterium Cluster EA Bacteriophages: Phylogenomic Relationships and Host Range Predictions
Source: Microorganisms. 2023 Jan 10;11(1):170. doi: 10.3390/microorganisms11010170 (PMC9863963; doi:10.3390/microorganisms11010170)
Supplement: Supplementary file 1 [file microorganisms-11-00170-s001.zip › microorganisms-2098370-SuppTable.pdf]

Supplementary Table S1. *Microbacterium* cluster EA bacteriophages included in the comparative analyses.

phage	sub-cluster	host	NJ-tree group[1]	length (in bp)	GC-content	# ORFs	# tRNAs	accession #	reference
AlexAdler	EA1	*M. foliorum* NRRL B-24224	3	41,834	63.4%	63	0	MG962360	Jacobs-Sera *et al.* 2020
Alyxandracam	EA1	*M. foliorum* NRRL B-24224	3	41,770	63.4%	63	0	MN062713	unpublished
Antoinette	EA1	*M. foliorum* NRRL B-24224	3	41,858	63.4%	63	0	MH045565	Jacobs-Sera *et al*. 2020
Aubergine	EA1	*M. foliorum* NRRL B-24224	5	41,555	63.4%	62	0	MG839015	Jacobs-Sera *et al.* 2020
AxiPup	EA1	*M. foliorum* NRRL B-24224	3	41,770	63.3%	63	0	MG839016	Jacobs-Sera *et al.* 2020
Baines	EA1	*M. foliorum* NRRL B-24224	single	41,555	63.4%	62	0	MG839017	Jacobs-Sera *et al.* 2020
Balsa	EA1	*M. foliorum* NRRL B-24224	2	41,862	63.4%	62	0	MG839030	Jacobs-Sera *et al.* 2020
Bandik	EA1	*M. foliorum* NRRL B-24224	3	41,804	63.5%	63	0	MH045554	Jacobs-Sera *et al.* 2020
BeautPeep30	EA1	*M. foliorum* NRRL B-24224	3	41,809	63.5%	63	0	MT310861	unpublished
BeeBee8	EA1	*M. foliorum* NRRL B-24224	2	42,027	63.3%	65	0	MH045555	Jacobs-Sera *et al.* 2020
Benjalauren	EA1	*M. foliorum* NRRL B-24224	4	41,561	63.5%	63	0	MN369737	unpublished
BigRedClifford	EA1	*M. foliorum* NRRL B-24224	3	41,797	63.3%	63	0	MT498049	unpublished
BonesMcCoy	EA1	*M. foliorum* NRRL B-24224	3	41,797	63.4%	63	0	MK967391	unpublished
Bonino	EA1	*M. foliorum* NRRL B-24224	5	41,534	63.4%	62	0	MG920061	Jacobs-Sera *et al.* 2020
Calix	EA1	*M. foliorum* NRRL B-24224	single	41,555	63.4%	62	0	MN234163	unpublished
Chako	EA1	*M. foliorum* NRRL B-24224	4	41,550	63.4%	62	0	OP867023	Milhaven *et al.*
Chamuel	EA1	*M. foliorum* NRRL B-24224	6	41,568	63.3%	62	0	MN807246	unpublished
Christoph	EA1	*M. foliorum* NRRL B-24224	5	41,554	63.4%	62	0	MN234222	unpublished
Clancy	EA1	*M. foliorum* NRRL B-24224	4	41,555	63.4%	62	0	MK967400	unpublished
Convict	EA1	*M. foliorum* NRRL B-24224	3	41,798	63.4%	63	0	MT310873	unpublished

Dave	EA1	*M. foliorum* NRRL B-24224	3	41,858	63.4%	63	0	MH045558	Jacobs-Sera *et al.* 2020
Den3	EA1	*M. foliorum* NRRL B-24224	3	41,800	63.4%	63	0	MN062702	unpublished
Dothraki	EA1	*M. foliorum* NRRL B-24224	3	41,809	63.6%	63	0	MT451985	unpublished
Erla	EA1	*M. foliorum* NRRL B-24224	7	41,538	63.4%	62	0	MW291026	Milhaven *et al*. 2021
Espinosa	EA1	*M. foliorum* NRRL B-24224	5	41,553	63.4%	62	0	MG839018	Jacobs-Sera *et al.* 2020
Etna	EA1	*M. foliorum* NRRL B-24224	2	41,908	63.4%	64	0	MH045559	Jacobs-Sera *et al.* 2020
Etta	EA1	*M. foliorum* NRRL B-24224	7	41,542	63.3%	62	0	MK977697	unpublished
Gargoyle	EA1	*M. foliorum* NRRL B-24224	3	41,803	63.5%	63	0	MH153802	Jacobs-Sera *et al.* 2020
Gelo	EA1	*M. foliorum* NRRL B-24224	single	41,562	63.4%	62	0	MG962367	Jacobs-Sera *et al.* 2020
Gershwin	EA1	*M. foliorum* NRRL B-24224	5	41,555	63.4%	62	0	MN234177	unpublished
Greys	EA1	*M. foliorum* NRRL B-24224	3	41,797	63.4%	63	0	MK757447	unpublished
Gubbabump	EA1	*M. foliorum* NRRL B-24224	5	41,492	63.4%	62	0	MT986026	unpublished
Hamlet	EA1	*M. foliorum* NRRL B-24224	2	41,934	63.4%	63	0	MG839019	Jacobs-Sera *et al.* 2020
HanSolo	EA1	*M. foliorum* NRRL B-24224	4	41,516	63.4%	62	0	MK967394	unpublished
Ilzat	EA1	*M. foliorum* NRRL B-24224	4	41,525	63.5%	62	0	MG839029	Jacobs-Sera *et al.* 2020
Ioannes	EA1	*M. foliorum* NRRL B-24224	3	41,879	63.4%	63	0	MN735430	El Yaman *et al*. 2020
Jenos	EA1	*M. foliorum* NRRL B-24224	1	42,413	63.8%	64	0	MZ005681	unpublished
Kale	EA1	*M. foliorum* NRRL B-24224	5	41,558	63.4%	62	0	MG839020	Jacobs-Sera *et al.* 2020
Klimt	EA1	*M. foliorum* NRRL B-24224	6	41,573	63.4%	65	0	MN617840	unpublished
Knox	EA1	*M. foliorum* NRRL B-24224	3	41,797	63.3%	63	0	MG839021	Jacobs-Sera *et al.* 2020
Kurt1	EA1	*M. foliorum* NRRL B-24224	3	41,803	63.6%	63	0	MT553341	unpublished

Leafus	EA1	*M. liquefaciens* LMG 16120	2	42,000	63.4%	63	0	MT498062	Frost *et al*. 2021
Lovelyunicorn	EA1	*M. foliorum* NRRL B-24224	3	41,798	63.4%	63	0	MT553339	unpublished
Ludgate	EA1	*M. foliorum* NRRL B-24224	3	41,799	63.5%	63	0	MG839022	Jacobs-Sera *et al.* 2020
MaeLinda	EA1	*M. foliorum* NRRL B-24224	3	41,809	63.6%	63	0	MT952846	unpublished
ManRay	EA1	*M. foliorum* NRRL B-24224	5	41,555	63.4%	62	0	MN234204	unpublished
Martin	EA1	*M. foliorum* NRRL B-24224	3	41,812	63.5%	64	0	MH153805	Jacobs-Sera *et al.* 2020
McGalleon	EA1	*M. foliorum* NRRL B-24224	1	42,562	63.7%	63	0	MN062703	unpublished
MillyPhilly	EA1	*M. foliorum* NRRL B-24224	3	41,800	63.5%	63	0	MK620903	Nguyen Quach *et al.* 2019
MonChoix	EA1	*M. foliorum* NRRL B-24224	2	41,670	63.4%	63	0	MK894437	unpublished
Nagem	EA1	*M. foliorum* NRRL B-24224	3	41,846	63.5%	63	0	MH045560	Jacobs-Sera *et al.* 2020
Nattles	EA1	*M. foliorum* NRRL B-24224	single	41,544	63.4%	62	0	MG925352	Jacobs-Sera *et al.* 2020
Oats	EA1	*M. foliorum* NRRL B-24224	5	41,555	63.4%	62	0	MH153806	Jacobs-Sera *et al.* 2020
Oxtober96	EA1	*M. foliorum* NRRL B-24224	3	41,798	63.4%	64	0	MT024864	unpublished
Papafritta	EA1	*M. foliorum* NRRL B-24224	4	41,573	63.4%	62	0	MH513981	Jacobs-Sera *et al.* 2020
ParleG	EA1	*M. foliorum* NRRL B-24224	3	41,810	63.6%	63	0	MT553343	unpublished
Peep	EA1	*M. foliorum* NRRL B-24224	3	41,856	63.4%	63	0	MG839023	Jacobs-Sera *et al.* 2020
Peppino	EA1	*M. foliorum* NRRL B-24224	2	41,932	63.3%	63	0	MG839024	Jacobs-Sera *et al.* 2020
Pherferi	EA1	*M. foliorum* NRRL B-24224	3	41,803	63.6%	62	0	MT310866	unpublished
Phriends	EA1	*M. foliorum* NRRL B-24224	3	41,606	63.6%	62	0	MK620902	Nguyen Quach *et al.* 2019
Pocket	EA1	*M. foliorum* NRRL B-24224	5	41,583	63.4%	62	0	MN234181	unpublished
PuppyEggo	EA1	*M. foliorum* NRRL B-24224	3	41,803	63.3%	63	0	MG944219	Jacobs-Sera *et al.* 2020

Raccoon	EA1	*M. foliorum* NRRL B-24224	2	41,894	63.4%	63	0	MG839025	Jacobs-Sera *et al.* 2020
Raptor	EA1	*M. foliorum* NRRL B-24224	3	41,801	63.4%	63	0	MH045562	Jacobs-Sera *et al.* 2020
Redfield	EA1	*M. foliorum* NRRL B-24224	2	41,930	63.3%	63	0	MH479922	Jacobs-Sera *et al.* 2020
Renzie	EA1	*M. foliorum* NRRL B-24224	3	41,872	63.4%	63	0	MN735435	unpublished
Riyhil	EA1	*M. foliorum* NRRL B-24224	3	41,812	63.5%	63	0	MK620895	Nguyen Quach *et al.* 2019
Robinson	EA1	*M. foliorum* NRRL B-24224	3	41,874	63.5%	63	0	MH045563	Jacobs-Sera *et al.* 2020
Schnapsidee	EA1	*M. foliorum* NRRL B-24224	2	41,872	63.4%	63	0	MH590590	Jacobs-Sera *et al.* 2020
Sedgewig	EA1	*M. foliorum* NRRL B-24224	4	41,505	63.4%	62	0	MT310851	unpublished
Shee	EA1	*M. foliorum* NRRL B-24224	3	41,800	63.5%	63	0	MK919471	unpublished
SJay	EA1	*M. foliorum* NRRL B-24224	3	41,847	63.5%	63	0	MZ958749	unpublished
SonOfLevi	EA1	*M. foliorum* NRRL B-24224	6	41,573	63.4%	64	0	MN892483	unpublished
Stanktossa	EA1	*M. foliorum* NRRL B-24224	3	41,806	63.6%	62	0	MN062709	unpublished
StingRay	EA1	*M. foliorum* NRRL B-24224	single	41,597	63.5%	62	0	MG944222	Jacobs-Sera *et al.* 2020
Stormbreaker8	EA1	*M. foliorum* NRRL B-24224	2	41,751	63.4%	62	0	MZ171379	unpublished
Strathdee	EA1	*M. foliorum* NRRL B-24224	3	41,802	63.4%	63	0	MZ005684	unpublished
Superfresh	EA1	*M. foliorum* NRRL B-24224	3	41,860	63.4%	63	0	MG839026	Jacobs-Sera *et al.* 2020
TatarkaPM	EA1	*M. foliorum* NRRL B-24224	3	41,813	63.5%	63	0	MT771350	unpublished
Teagan	EA1	*M. foliorum* NRRL B-24224	3	41,793	63.5%	63	0	MH153811	Jacobs-Sera *et al.* 2020
TeddyBear	EA1	*M. foliorum* NRRL B-24224	5	41,555	63.4%	63	0	MH045564	Jacobs-Sera *et al.* 2020
Tenda	EA1	*M. foliorum* NRRL B-24224	5	41,553	63.4%	62	0	MG839028	Jacobs-Sera *et al.* 2020
Thorongil	EA1	*M. foliorum* NRRL B-24224	3	41,331	63.3%	63	0	MW055911	unpublished

TinSulphur	EA1	*M. foliorum* NRRL B-24224	3	41,807	63.5%	64	0	MK620905	Nguyen Quach *et al.* 2019
Velene	EA1	*M. foliorum* NRRL B-24224	3	41,798	63.4%	63	0	MK967388	unpublished
WildNOut	EA1	*M. foliorum* NRRL B-24224	3	41,796	63.4%	63	0	MW435859	unpublished
Winzigespinne	EA1	*M. foliorum* NRRL B-24224	5	41,556	63.4%	62	0	MT986027	unpublished
Zada	EA1	*M. foliorum* NRRL B-24224	3	41,814	63.5%	64	0	MT310856	El Yaman *et al*. 2020
Andromedas	EA2	*M. foliorum* NRRL B-24224		40,494	62.0%	63	0	MH590606	Jacobs-Sera *et al.* 2020
ColaCorta	EA2	*M. foliorum* NRRL B-24224		40,494	62.0%	63	0	MH590604	Jacobs-Sera *et al.* 2020
Eleri	EA2	*M. foliorum* NRRL B-24224		40,366	62.0%	63	0	MG839027	Jacobs-Sera *et al.* 2020
Finny	EA2	*M. foliorum* NRRL B-24224		40,313	62.1%	59	0	MK894432	Lee *et al*. 2019
Glamour	EA2	*M. foliorum* NRRL B-24224		40,224	62.1%	62	0	MT553340	unpublished
MCubed	EA2	*M. foliorum* NRRL B-24224		40,381	62.0%	62	0	MN096378	unpublished
Sansa	EA2	*M. foliorum* NRRL B-24224		40,306	61.8%	62	0	MH513982	Jacobs-Sera *et al.* 2020
Casey	EA3	*M. foliorum* NRRL B-24224		39,307	61.3%	59	0	MG944226	Jacobs-Sera *et al.* 2020
Pajaza	EA3	*M. foliorum* NRRL B-24224		39,307	61.2%	59	0	MG944216	Jacobs-Sera *et al.* 2020
Pikmin	EA3	*M. foliorum* NRRL B-24224		39,307	61.3%	59	0	MG944218	Jacobs-Sera *et al.* 2020
Bustleton	EA4	*M. foliorum* NRRL B-24224		39,184	64.4%	57	0	MK814762	Nguyen Quach *et al.* 2019
Golden	EA4	*M. foliorum* NRRL B-24224		39,640	64.1%	58	0	MG925343	Jacobs-Sera *et al.* 2020
Kauala	EA4	*M. foliorum* NRRL B-24224		39,378	64.4%	56	0	MT657344	Lin *et al.* 2020
Koji	EA4	*M. foliorum* NRRL B-24224		39,403	64.2%	56	0	MG925345	Jacobs-Sera *et al.* 2020
Lucky3	EA4	*M. foliorum* NRRL B-24224		39,640	64.1%	58	0	MG925347	Jacobs-Sera *et al.* 2020
Pherbot	EA4	*M. foliorum* NRRL B-24224		39,171	64.4%	57	0	MK620904	Nguyen Quach *et al.* 2019

PrincePhergus	EA4	*M. foliorum* NRRL B-24224	39,191	64.6%	57	0	MK620901	Nguyen Quach *et al.* 2019
Sinatra	EA4	*M. foliorum* NRRL B-24224	39,183	64.4%	58	0	MK937602	Jacobs-Sera *et al.* 2020
Alakazam	EA5	*M. foliorum* NRRL B-24224	41,269	64.4%	60	0	MT024862	unpublished
Hasitha	EA5	*M. foliorum* NRRL B-24224	39,952	63.6%	62	0	MZ622182	unpublished
Nebulous	EA5	*M. liquefaciens* LMG 16120	41,419	64.3%	57	0	MT451984	Frost *et al*. 2021
Neferthena	EA5	*M. foliorum* NRRL B-24224	41,706	64.4%	63	0	MH697589	Jacobs-Sera *et al.* 2020
QuadZero	EA5	*M. foliorum* NRRL B-24224	40,140	63.6%	62	0	MZ150787	Cook-Easterwood *et al*. 2022
Zayuliv	EA5	*M. foliorum* NRRL B-24224	40,457	63.9%	62	0	MT889377	unpublished
Zepp	EA5	*M. foliorum* NRRL B-24224	40,189	63.6%	62	0	MN908686	unpublished
Chepli	EA6	*M. foliorum* NRRL B-24224	40,332	63.0%	64	0	MK875794	Jacobs-Sera *et al.* 2020
Juicer	EA6	*M. foliorum* NRRL B-24224	41,099	62.8%	63	0	MZ622175	unpublished
KatChan	EA6	*M. foliorum* NRRL B-24224	40,349	63.0%	63	0	MZ820084	unpublished
WilliamStrong	EA6	*M. paraoxydans* NRRL B-14843	40,613	63.7%	63	0	MZ355727	unpublished
Theresita	EA7	*M. natoriense* ATCC BAA-1032	40,234	65.9%	57	0	MK660713	Jacobs-Sera *et al.* 2020
Schubert	EA8	*M. foliorum* NRRL B-24224	38,820	61.4%	56	0	MK308637	Jacobs-Sera *et al.* 2020
ChickenKing	EA9	*M. foliorum* NRRL B-24224	39,924	63.4%	57	0	MN310543	unpublished
GaeCeo	EA9	*M. foliorum* NRRL B-24224	40,168	63.4%	60	0	MT657343	unpublished
Mandalorian	EA10	*M. foliorum* NRRL B-24224	40,407	63.8%	63	0	MZ005670	unpublished
Nucci	EA10	*M. foliorum* NRRL B-24224	40,373	63.7%	63	0	MN096367	Kim *et al*. 2022
YuuY	EA10	*M. foliorum* NRRL B-24224	40,996	63.2%	64	2	MN284901	Kim *et al*. 2022
Ixel	EA11	*M. liquefaciens* LMG 16120	40,556	63.3%	61	0	MT451983	Frost *et al*. 2021

[1] NJ-tree group = sub-cluster EA1 groupings according to the neighbor-joining (NJ) tree in Figure 1

Supplementary Table S2. *Microbacteria* hosts included in the comparative analyses.

host	# genes	length (kb)	GC-content	accession #	reference
M. amylolyticum	2,504	2,543	64.6%	NZ_CP049253.1	Anand *et al.* 2012
M. aurum	3,343	3,425	69.9%	NZ_CP018762.1	Yokota *et al.* 1993
M. chengjingii	3,495	3,623	61.9%	NZ_CP061169.1	Zhou *et al.* 2021
M. endophyticum	2,756	2,916	61.8%	NZ_CP049255.1	Alves *et al.* 2014
M. fandaimingii	3,506	3,622	63.3%	NZ_CP061170.1	Zhou *et al.* 2021
M. foliorum	3,379	3,568	68.7%	NZ_CP031425.1	Russell *et al.* 2019
M. liquefaciens	3,651	3,712	68.3%	NZ_CP101471.1	Gómez-Ramírez *et al.* 2015
M. lushaniae	3,550	3,650	70.7%	NZ_CP044232.1	Tian *et al.* 2021
M. luteum	3,418	3,520	69.2%	NZ_CP063814.1	Xie *et al.* 2021
M. oleivorans	2,907	2,997	69.0%	CP031421.1	Ortet *et al.* 2017
M. paraoxydans	3,565	3,415	69.9%	NZ_CP064873.1	Laffineur *et al.* 2003
M. protaetiae	3,555	3,790	68.0%	NZ_CP035494.1	Heo *et al.* 2020
M. resistens	3,802	4,011	71.3%	NZ_CP082781.1	Behrendt *et al.* 2001
M. wangchenii	3,743	3,828	70.6%	NZ_CP038266.1	Dong *et al.* 2019

Supplementary Table S3. Matrix of ANI values of representative cluster EA bacteriophages used in the dot plot analysis (Supplementary Figure S1).

	McGalleon (EA1.1)	Hamlet (EA1.2)	Sjay (EA1.3)	StingRay (EA1)	Papafritta (EA1.4)	Oats (EA1.5)	Baines (EA1)	Nattles (EA1)	SonOfLevi (EA1.6)	Gelo (EA1)	Erla (EA1.7)
McGalleon (EA1.1)	1.0000	0.8108	0.8092	0.8096	0.8094	0.8092	0.8094	0.8088	0.8086	0.8093	0.8088
Hamlet (EA1.2)	0.8108	1.0000	0.8240	0.8249	0.8236	0.8260	0.8286	0.8282	0.8281	0.8276	0.8280
Sjay (EA1.3)	0.8092	0.8240	1.0000	0.9672	0.9607	0.9640	0.9579	0.9538	0.9535	0.9546	0.9544
StingRay (EA1)	0.8096	0.8249	0.9672	1.0000	0.9879	0.9809	0.9681	0.9637	0.9626	0.9633	0.9634
Papafritta (EA1.4)	0.8094	0.8236	0.9607	0.9879	1.0000	0.9753	0.9617	0.9573	0.9566	0.9564	0.9566
Oats (EA1.5)	0.8092	0.8260	0.9640	0.9809	0.9753	1.0000	0.9650	0.9654	0.9620	0.9636	0.9636
Baines (EA1)	0.8094	0.8286	0.9579	0.9681	0.9617	0.9650	1.0000	0.9875	0.9850	0.9871	0.9867
Nattles (EA1)	0.8088	0.8282	0.9538	0.9637	0.9573	0.9654	0.9875	1.0000	0.9922	0.9941	0.9938
SonOfLevi (EA1.6)	0.8086	0.8281	0.9535	0.9626	0.9566	0.9620	0.9850	0.9922	1.0000	0.9942	0.9943
Gelo (EA1)	0.8093	0.8276	0.9546	0.9633	0.9564	0.9636	0.9871	0.9941	0.9942	1.0000	0.9962
Erla (EA1.7)	0.8088	0.8280	0.9544	0.9634	0.9566	0.9636	0.9867	0.9938	0.9943	0.9962	1.0000
Finny (EA2)	0.7058	0.7003	0.7084	0.7093	0.7085	0.7094	0.7095	0.7101	0.7096	0.7097	0.7100
Pajaza (EA3)	0.6861	0.6806	0.6829	0.6879	0.6859	0.6846	0.6864	0.6880	0.6868	0.6877	0.6879
Koji (EA4)	0.7097	0.7099	0.7144	0.7147	0.7154	0.7135	0.7136	0.7144	0.7152	0.7150	0.7151
QuadZero (EA5)	0.6743	0.6812	0.6806	0.6832	0.6787	0.6842	0.6832	0.6824	0.6815	0.6821	0.6825
Juicer (EA6)	0.6911	0.6893	0.6833	0.6854	0.6826	0.6834	0.6817	0.6821	0.6813	0.6809	0.6817
Theresita (EA7)	0.6748	0.6762	0.6683	0.6799	0.6736	0.6750	0.6731	0.6756	0.6756	0.6786	0.6738
Schubert (EA8)	0.7027	0.7020	0.7054	0.7067	0.7053	0.7055	0.7061	0.7079	0.7081	0.7058	0.7060

GaeCeo (EA9)	0.7234	0.7210	0.7266	0.7272	0.7256	0.7259	0.7271	0.7253	0.7256	0.7262	0.7258
Nucci (EA10)	0.7057	0.7042	0.7103	0.7122	0.7115	0.7121	0.7129	0.7123	0.7119	0.7123	0.7125
Ixel (EA11)	0.6930	0.6898	0.6936	0.6963	0.6960	0.6967	0.6949	0.6941	0.6939	0.6937	0.6939

Supplementary Table S3 cont.

	Finny (EA2)	Pajaza (EA3)	Koji (EA4)	QuadZero (EA5)	Juicer (EA6)	Theresita (EA7)	Schubert (EA8)	GaeCeo (EA9)	Nucci (EA10)	Ixel (EA11)
McGalleon (EA1.1)	0.7058	0.6861	0.7097	0.6743	0.6911	0.6748	0.7027	0.7234	0.7057	0.6930
Hamlet (EA1.2)	0.7003	0.6806	0.7099	0.6812	0.6893	0.6762	0.7020	0.7210	0.7042	0.6898
Sjay (EA1.3)	0.7084	0.6829	0.7144	0.6806	0.6833	0.6683	0.7054	0.7266	0.7103	0.6936
StingRay (EA1)	0.7093	0.6879	0.7147	0.6832	0.6854	0.6799	0.7067	0.7272	0.7122	0.6963
Papafritta (EA1.4)	0.7085	0.6859	0.7154	0.6787	0.6826	0.6736	0.7053	0.7256	0.7115	0.6960
Oats (EA1.5)	0.7094	0.6846	0.7135	0.6842	0.6834	0.6750	0.7055	0.7259	0.7121	0.6967
Baines (EA1)	0.7095	0.6864	0.7136	0.6832	0.6817	0.6731	0.7061	0.7271	0.7129	0.6949
Nattles (EA1)	0.7101	0.6880	0.7144	0.6824	0.6821	0.6756	0.7079	0.7253	0.7123	0.6941
SonOfLevi (EA1.6)	0.7096	0.6868	0.7152	0.6815	0.6813	0.6756	0.7081	0.7256	0.7119	0.6939
Gelo (EA1)	0.7097	0.6877	0.7150	0.6821	0.6809	0.6786	0.7058	0.7262	0.7123	0.6937
Erla (EA1.7)	0.7100	0.6879	0.7151	0.6825	0.6817	0.6738	0.7060	0.7258	0.7125	0.6939
Finny (EA2)	1.0000	0.6999	0.7259	0.6924	0.7079	0.6927	0.7137	0.7264	0.7605	0.7118
Pajaza (EA3)	0.6999	1.0000	0.7096	0.6662	0.6895	0.6669	0.7213	0.7010	0.7001	0.6738
Koji (EA4)	0.7259	0.7096	1.0000	0.6961	0.6992	0.7107	0.7221	0.7540	0.7374	0.6934
QuadZero (EA5)	0.6924	0.6662	0.6961	1.0000	0.7029	0.6478	0.6809	0.6866	0.7129	0.7084
Juicer (EA6)	0.7079	0.6895	0.6992	0.7029	1.0000	0.6390	0.6914	0.6991	0.7087	0.7124
Theresita (EA7)	0.6927	0.6669	0.7107	0.6478	0.6390	1.0000	0.6854	0.6998	0.7001	0.6375
Schubert (EA8)	0.7137	0.7213	0.7221	0.6809	0.6914	0.6854	1.0000	0.7275	0.7128	0.6982

GaeCeo (EA9)	0.7264	0.7010	0.7540	0.6866	0.6991	0.6998	0.7275	1.0000	0.7163	0.7018
Nucci (EA10)	0.7605	0.7001	0.7374	0.7129	0.7087	0.7001	0.7128	0.7163	1.0000	0.7117
Ixel (EA11)	0.7118	0.6738	0.6934	0.7084	0.7124	0.6375	0.6982	0.7018	0.7117	1.0000

Supplementary Table S4. Exploratory host range prediction. Putative host genera of the 125 cluster EA bacteriophages included in this study as predicted by PHERI (Baláž et al., 2020).

phage	sub-cluster	*Microbacterium*	*Burkholderia*	*Mycolicibacterium*	*Aeromonas*	*Acinetobacter*	*Cellulophaga*	*Synechochoccus*	*Mycobacterium*	*Lactococcus*	*Rhizobium*	*Gordonia*
AlexAdler	EA1	✓										
Alyxandracam	EA1	✓										
Antoinette	EA1	✓										
Aubergine	EA1	✓										
AxiPup	EA1	✓										
Baines	EA1	✓										
Balsa	EA1	✓										
Bandik	EA1	✓										
BeautPeep30	EA1	✓										
BeeBee8	EA1											
Benjalauren	EA1	✓										
BigRedClifford	EA1	✓										
BonesMcCoy	EA1	✓										
Bonino	EA1	✓										
Calix	EA1	✓										
Chako	EA1	✓										
Chamuel	EA1	✓										
Christoph	EA1	✓										
Clancy	EA1	✓										

Convict	EA1	✓
Dave	EA1	✓
Den3	EA1	✓
Dothraki	EA1	✓
Erla	EA1	✓
Espinosa	EA1	✓
Etna	EA1	✓
Etta	EA1	✓
Gargoyle	EA1	✓
Gelo	EA1	✓
Gershwin	EA1	✓
Greys	EA1	✓
Gubbabump	EA1	✓
Hamlet	EA1	✓
HanSolo	EA1	✓
Ilzat	EA1	✓
Ioannes	EA1	✓
Jenos	EA1	✓
Kale	EA1	✓
Klimt	EA1	✓
Knox	EA1	✓
Kurt1	EA1	✓
Leafus	EA1	✓
Lovelyunicorn	EA1	✓
Ludgate	EA1	✓

MaeLinda	EA1	✓
ManRay	EA1	✓
Martin	EA1	✓
McGalleon	EA1	✓
MillyPhilly	EA1	✓
MonChoix	EA1	✓
Nagem	EA1	✓
Nattles	EA1	✓
Oats	EA1	✓
Oxtober96	EA1	✓
Papafritta	EA1	✓
ParleG	EA1	✓
Peep	EA1	✓
Peppino	EA1	✓
Pherferi	EA1	✓
Phriends	EA1	✓
Pocket	EA1	✓
PuppyEggo	EA1	✓
Raccoon	EA1	✓
Raptor	EA1	✓
Redfield	EA1	✓
Renzie	EA1	✓
Riyhil	EA1	✓
Robinson	EA1	✓
Schnapsidee	EA1	✓

Sedgewig	EA1	✓	
Shee	EA1	✓	
SJay	EA1	✓	
SonOfLevi	EA1	✓	
Stanktossa	EA1	✓	
StingRay	EA1	✓	
Stormbreaker8	EA1	✓	
Strathdee	EA1	✓	
Superfresh	EA1	✓	
TatarkaPM	EA1	✓	
Teagan	EA1	✓	
TeddyBear	EA1	✓	
Tenda	EA1	✓	
Thorongil	EA1	✓	
TinSulphur	EA1	✓	
Velene	EA1	✓	
WildNOut	EA1	✓	
Winzigespinne	EA1	✓	
Zada	EA1	✓	
Andromedas	EA2		
ColaCorta	EA2		
Eleri	EA2		
Finny	EA2	✓	✓
Glamour	EA2		
MCubed	EA2		

Sansa	EA2	✓		✓							
Casey	EA3	✓									
Pajaza	EA3	✓									
Pikmin	EA3	✓									
Bustleton	EA4	✓			✓	✓					
Golden	EA4	✓									
Kauala	EA4	✓									
Koji	EA4	✓									
Lucky3	EA4	✓									
Pherbot	EA4	✓			✓	✓					
PrincePhergus	EA4	✓									
Sinatra	EA4	✓									
Alakazam	EA5	✓									
Hasitha	EA5						✓				
Nebulous	EA5	✓									
Neferthena	EA5	✓					✓				
QuadZero	EA5	✓						✓			
Zayuliv	EA5	✓									
Zepp	EA5	✓	✓	✓							
Chepli	EA6		✓								
Juicer	EA6										
KatChan	EA6		✓								
WilliamStrong	EA6	✓							✓	✓	
Theresita	EA7							✓			
Schubert	EA8	✓									✓

ChickenKing	EA9	✓			✓
GaeCeo	EA9	✓			
Mandalorian	EA10	✓		✓	
Nucci	EA10			✓	
YuuY	EA10	✓			
Ixel	EA11	✓	✓		

REFERENCES

Alves, A.; Correia, A.; Igual, J.M.; Trujillo, M.E. *Microbacterium endophyticum* sp. nov. and *Microbacterium halimionae* sp. nov., endophytes isolated from the salt-marsh plant *Halimione portulacoides* and emended description of the genus *Microbacterium*. *Sys. Appl. Microbiol*. **2014**, *37*, 474–479.

Anand, S.; Bala, K.; Saxena, A.; Schumann, P.; Lal, R. *Microbacterium amylolyticum* sp. nov., isolated from soil from an industrial waste site. *Int. J. Syst. Evol. Microbiol.* **2012**, *62*, 2114–2120.

Baláž, A.; Kajsík, M.; Budiš, J.; Szemeš, T.; Turňa, J. PHERI—Phage Host Exploration Pipeline. bioRxiv 2020. Available online: https://www.biorxiv.org/content/10.1101/2020.05.13.093773v3 (accessed on 20 December 2022)

Behrendt, U.; Ulrich, A.; Schumann, P. Description of *Microbacterium foliorum* sp. nov. and *Microbacterium phyllosphaerae* sp. nov., isolated from the phyllosphere of grasses and the surface litter after mulching the sward, and reclassification of *Aureobacterium resistens* (Funke *et al.* 1998) as *Microbacterium resistens* comb. nov. *Int. J. Syst. Evol. Microbiol.* **2001**, *51*, 1267–1276.

Cook-Easterwood, J.; Bethel, D.J.; Kurikose, I.; Katsanos, J. The genomic characterization of two *Microbacterium foliorum*–specific bacteriophages, QuadZero and AnnaLie. *Microbiol. Resour. Announc*. **2022**, *11*, e00208-22.

Dong, K.; Yang, J.; Lu, S.; Pu, J.; Lai, X.-H.; Jin, D.; Li, J.; Zhang, G.; Wang, X.; Zhang, S.; et al. *Microbacterium wangchenii* sp. nov., isolated from faeces of Tibetan gazelles (*Procapra picticaudata*) on the Qinghai-Tibet plateau. *Int. J. Syst. Evol. Microbiol.* **2019**, *70*, 1307–1314.

El Yaman, R.; Anderson, J.S.; Anderson, T.M.; Avalos, M.A.; Bangurah, S.K.; Dada, K.B.; Degener, K.R.; Hadeed, M.N.; Issa, L.H.; Jaeran, A.S.; et al. Genome sequences of microbacteriophages Zada and Ioannes. *Microbiol. Resour. Announc*. **2020**, *9*, e01012-20.

Frost, V.J.; Westover, K.M. Complete genome sequences of *Microbacterium liquefaciens* phages Mercedes, Leafus, Nebulous, and Ixel. *Microbiol. Resour. Announc*. **2021**, *10*, e00068-21.

Gómez-Ramírez, M.; Montero-Álvarez, L.A.; Tobón-Avilés, A.; Fierros-Romero, G.; Rojas-Avelizapa, N.G. *Microbacterium oxydans* and *Microbacterium liquefaciens*: A biological alternative for the treatment of Ni-V-containing wastes. *J. Environ. Sci. Health A Tox. Hazard Subst. Environ. Eng.* **2015**, *50*, 602–610.

Heo, J.; Cho, H.; Kim, M.A.; Hamada, M.; Tamura, T.; Saitou, S.; Kim, S.-J.K.; Kwon, S.-W. 2020 *Microbacterium protaetiae* sp. nov. isolated from gut of larva of *Protaetia brevitarsis seulensis*. *Int. J. Syst. Evol. Microbiol.* **2020**, *70*, 2226–2232.

Jacobs-Sera, D.; Abad, L.A.; Alvey, R.M.; Anders, K.R.; Aull, H.G.; Bhalla, S.S.; Blumer, L.S.; Bollivar, D.W.; Bonilla, J.A.; Butela, K.A.; et al. Genomic diversity of bacteriophages infecting *Microbacterium* spp. *PLoS One* **2020**, *15*, e0234636.

Kim, U.; Paul, E.S.; Diaz, A. Characterization of phages YuuY, KaiHaiDragon, and OneinaGillian isolated from *Microbacterium foliorum*. *Int. J. Mol. Sci.* **2022**, *23*, 6609.

Laffineur, K.; Avesani, V.; Cornu, G.; Charlier, J.; Janssens, M.; Wauters, G.; Delmée, M. Bacteremia due to a novel *Microbacterium* species in a patient with leukemia and description of *Microbacterium paraoxydans* sp. nov. *J. Clin. Microbiol.* **2003**, *41*, 2242–2246.

Lee, T.; Aguirre, M.; Andrews, S.; Bahr, K.; Ballard, A.; Bristerpostma, M.; Cox, F.; Dowell, L.; Kiker, D.; Lujan, T.; et al. Complete genome sequence of bacteriophage Finny, isolated from a *Microbacterium foliorum* culture. *Microbiol. Resour. Announc*. **2019**, *8*, e01039-19.

Lin, H.; Reeves, M.; Acevedo, M.; Bass, K.; Chau, E.; Ching, B.; Enriquez, E.; Evans, S.; Mamora, K.; Pang, C.; et al. Genome sequences of bacteriophages ClearAsMud and Kauala, isolated from *Microbacterium foliorum*. *Microbiol. Resour. Announc*. **2020**, *9*, e01026-20.

Milhaven, M.; Cai, L; Cherian, S.; Johnson, K.; Salas Perez, K.; Blanco, M.; Garg, A.; Lobatos, J.; Mitra, C.; Strasser, M.; Harms, R.; Pfeifer, S.P. Complete genome sequence of the *Microbacterium* bacteriophage Chako. *Microbiol. Resour. Announc*., in review.

Milhaven, M.; Hastings, E.; Brister, D.; Cevallos, L.; Chilukuri, S.; Diyya, A.; Garg, A.; Hernandez, R.; Kelly, D.; Lazo, K.; et al. Complete genome sequence of *Microbacterium* bacteriophage Erla. *Microbiol. Resour. Announc*. **2021**, *10*, e01354-20.

Nguyen Quach, A.M.; Varghese, A.E.; Siddiq, K.; Pappano, I.G.; Thaha, A.; Marcelis, T.A.; Feruku, B.; Vindyala, A.; Mohamed, R.; Johnson, K.A.; et al. Complete genome sequences of seven EA cluster microbacteriophages, Bustleton, MillyPhilly, Riyhil, Phriends, Pherbot, PrincePhergus, and TinSulphur. *Microbiol. Resour. Announc*. **2019**, *8*, e01193-19.

Ortet, P.; Gallois, N.; Piette, L.; Long, J.; Berthomieu, C.; Armengaud, J.; Barakat, M.; Chapon, V. Draft genome sequence of *Microbacterium oleivorans* strain A9, a bacterium isolated from Chernobyl radionuclide-contaminated soil. *Genome Announc.* **2017**, *5*, e00092-17.

Russell, D.A.; Garlena, R.A.; Hatfull, G.F. Complete genome sequence of *Microbacterium foliorum* NRRL B-24224, a host for bacteriophage discovery. *Microbiol. Resour. Announc*. **2019**, *8*, e01467-18.
Tian, Z.; Yang, J.; Lai, X.-H.; Pu, J.; Jin, D.; Luo, X.; Huang, Y.; Li, J.; Zhang, G.; Wang, S.; et al. *Microbacterium caowuchunii* sp. nov. and *Microbacterium lushaniae* sp. nov., isolated from Plateau Pika (*Ochotona Curzoniae*) on the Qinghai–Tibet plateau of PR China. *Int. J. Syst. Evol. Microbiol.* **2021**, *71*, 004662.
Xie, F.; Niu, S.; Lin, X.; Pei, S.; Jiang, L.; Tian, Y.; Zhang, G. Description of *Microbacterium* luteum sp. nov., *Microbacterium cremeum* sp. nov., and *Microbacterium atlanticum* sp. nov., three novel C50 carotenoid producing bacteria. *J. Microbiol*. **2021**, *59*, 886–897.
Yokota, A.; Takeuchi, M.; Weiss, N. Proposal of two new species in the genus *Microbacterium*: *Microbacterium dextranolyticum* sp. nov. and *Microbacterium Aurum* sp. nov. *Int. J. Syst. Evol. Microbiol.* **1993**, *43*, 549–554.
Zhou, J.; Huang, Y.; Yang, J.; Lai, X.-H.; Jin, D.; Lu, S.; Pu, J.; Zhang, S.; Zhu, W.; Xu, M.; et al. *Microbacterium chengjingii* sp. nov. and *Microbacterium fandaimingii* sp. nov., isolated from bat faeces of *Hipposideros* and *Rousettus* species. *Int. J. Syst. Evol. Microbiol.* **2021**, *71*, 004858.
